# Integrated Single-Nucleus and Epigenomic Profiling Reveals Coordinated Neuro-Glial and Chromatin Remodeling in the Type 2 Diabetic Hypothalamus

**DOI:** 10.3390/cells15181672

**Published:** 2026-09-16

**Authors:** Shuoyang Zhao, Shengwei Sui, Xinghui Guo, Xiaofeng Ji, Tingting Liu, Hongbo Yang

**Affiliations:** 1 Institute of Reproduction and Development, Shanghai Key Laboratory of Reproduction and Development, Obstetrics and Gynecology Hospital, Fudan University, Shanghai 200030, China; 20210880008@fudan.edu.cn (S.Z.); 23111250001@m.fudan.edu.cn (S.S.); 2 School of Public Health, State Key Laboratory of Digital Medical Engineering, Advanced Institute for Life and Health, Southeast University, Nanjing 210009, China; guoxinghui2018@163.com (X.G.); 220244223@seu.edu.cn (X.J.); 3 Shanghai Key Laboratory of Female Reproductive Endocrine Related Diseases, Shanghai 200090, China

**Keywords:** type 2 diabetes, hypothalamus, single-nucleus RNA-seq, ATAC-seq, neuropeptide Y, oligodendrocyte lineage, *Cntnap2*, neuro-glial interactions

## Abstract

**Highlights:**

**What are the main findings?**

• Integrated single-nucleus transcriptomic and tissue-level chromatin accessibility profiling identifies coordinated neuronal, oligodendrocyte-lineage, and regulatory remodeling in the hypothalamus of an HFD/STZ-induced T2D mouse model.
• Multi-omic evidence highlights activation of the *Npy* regulatory axis and identifies an increased *Cntnap2*-positive oligodendrocyte-lineage transcriptional state associated with altered predicted neuron–glia communication.

**What are the implications of the main findings?**

• The study extends the cellular landscape of diabetes-associated hypothalamic remodeling beyond neuronal populations by implicating oligodendrocyte-lineage states and neuro–glial signaling networks.
• The *Npy*-associated transcriptional and chromatin changes and the *Cntnap2*-positive oligodendrocyte-lineage state provide testable molecular and cellular features for future studies of hypothalamic metabolic regulation and neuron–glia interactions in diabetes.

**Abstract:**

The hypothalamus is a central regulator of systemic energy balance and glucose homeostasis, yet how type 2 diabetes (T2D) alters its cellular and regulatory landscape remains incompletely understood. Here, we integrated single-nucleus RNA sequencing (snRNA-seq) with bulk ATAC-seq to characterize hypothalamic remodeling in an HFD/STZ-induced T2D mouse model. High-resolution snRNA-seq revealed pronounced cell type-specific alterations involving several neuronal subpopulations, including Gal.Nts.Npy, Nxph4.Adcyap1, and Lmx1a.Gpr149.Tcf4 neurons, together with substantial changes in oligodendrocyte-lineage cells. Increased *Npy* expression was accompanied by greater tissue-level chromatin accessibility at the *Npy* locus, providing convergent evidence for activation of the hypothalamic *Npy* regulatory axis. Pseudotime analysis further showed increased representation of a transcriptionally distinct *Cntnap2*-positive oligodendrocyte-lineage state, together with reduced representation of canonical myelination-associated states. Ligand–receptor analysis predicted reduced signaling across selected neuron–oligodendrocyte interactions and altered communication among glial populations. Collectively, these findings reveal coordinated neuronal, oligodendrocyte-lineage, intercellular signaling, and chromatin-accessibility alterations in the diabetic hypothalamus, and identify the *Npy* regulatory axis and *Cntnap2*-positive oligodendrocyte-lineage state as candidate features for further mechanistic investigation.

## 1. Introduction

The hypothalamus integrates circulating hormonal and nutrient signals with neural inputs to coordinate feeding [1], energy expenditure [2], glucose homeostasis [3], and neuroendocrine and autonomic outputs [4]. Type 2 diabetes mellitus (T2D) is increasingly recognized not only as a peripheral metabolic disorder but also as a systemic disease with substantial effects on the nervous system, including central insulin resistance, neuroinflammation, autonomic dysfunction, and cognitive impairment [5,6]. Substantial epidemiological evidence links diabetes to an increased risk of cognitive decline and dementia [7,8,9]. As a central regulator of systemic metabolism, the hypothalamus is particularly vulnerable to chronic nutrient excess and the associated hormonal, metabolic, and inflammatory disturbances that accompany obesity and T2D [10]. Owing to its pronounced anatomical and cellular heterogeneity, these pathological effects are likely to differ across hypothalamic nuclei and cell populations [11,12,13].

Recent cell-resolved studies have begun to reveal the complexity of hypothalamic responses to metabolic disease. Single-nucleus and spatial transcriptomic analyses of obese and diabetic macaques identified region- and cell-type-specific hypothalamic dysfunction, particularly within the infundibular and paraventricular nuclei, involving tanycytic barrier disruption, microglial activation, neuronal inflammation, and impaired metabolic and neuronal activity programs [13]. In mice, prolonged diet-induced obesity selectively altered leptin- and CREB-associated signaling in agouti-related peptide-expressing neurons [14] whereas recurrent hypoglycemia in diabetic animals induced widespread transcriptional changes across multiple hypothalamic cell types despite largely preserved major-cell-type composition [15]. Together, these findings suggest that metabolic stress induces broad, coordinated remodeling of neuronal and glial states in the hypothalamus, extending beyond canonical glucose- and appetite-sensing circuits.

In addition to transcriptional alterations, metabolic disease can reshape the hypothalamic regulatory landscape [16]. Multi-omic profiling of dietary obesity demonstrated that high-fat-diet feeding modifies histone marks, DNA methylation, and chromatin accessibility in the mouse hypothalamus [16]. Notably, chromatin accessibility changes were cell-type-dependent, with excitatory neurons, inhibitory neurons, and oligodendrocytes among the most prominently affected populations. These findings suggest that altered chromatin regulation may contribute to the establishment or maintenance of disease-associated neuronal and glial transcriptional states [16].

Despite these advances, previous studies have mainly focused on isolated hypothalamic nuclei in diet-induced obesity, spatially resolved changes in non-human primates, epigenetic remodeling induced by high-fat feeding, or adaptations to recurrent hypoglycemia in diabetic animals [13,14,15,16]. Consequently, it remains unclear how established T2D alters the cellular composition and transcriptional states of the mouse hypothalamus and whether these changes are accompanied by corresponding alterations in chromatin accessibility. In particular, the chromatin-accessibility landscape of the hypothalamus in experimental T2D remains insufficiently characterized.

## 2. Materials and Methods

### 2.1. Mouse Models

All animal experiments received ethical clearance from the Shanghai model organisms center, IACUC (Approval No. 2024-0009), and were carried out in strict compliance with institutional policies. The C57BL/6 mice were kept under pathogen-free housing conditions (five per cage, 24 °C ambient temperature, 12-h light/dark cycle), with unrestricted access to food and water. Upon arrival at the vivarium, the subjects underwent a mandatory one-week acclimation period before being randomly distributed across the experimental cohorts to minimize potential bias. Trained staff monitored the physical condition of the animals two to three times weekly, and no symptoms of disease were noted in any individual throughout the study. All possible precautions were implemented to alleviate discomfort and distress.

### 2.2. Type 2 Diabetes Mellitus Model Induction

Female C57BL/6 mice (8–10 weeks old, weighing 15–20 g) were housed under controlled environmental conditions (24 ± 1 °C, 55 ± 5% humidity, and a 12-h light/dark cycle) with free access to food and water. After 5 days of acclimation, animals were randomly assigned to control and experimental groups. To establish a high-fat diet and STZ-induced diabetic model, mice in the experimental group were fed a high-fat diet (HFD; 60% fat by energy, D12492, Research Diets) for 3 weeks, while control animals received a low-fat chow diet. On day 22, following a 6–8 h fast, experimental mice received 4 consecutive daily injections of streptozotocin (STZ; 40 mg/kg; Sigma-Aldrich) freshly dissolved in cold 50 mM sodium citrate buffer (pH = 4.5). Control animals received an equivalent volume of sodium citrate buffer alone. Mice were subsequently maintained on their respective diets. Four days after the final STZ administration, fasting blood glucose levels were measured from tail-vein samples using a glucometer. Mice exhibiting fasting blood glucose concentrations > 15 mmol/L (270 mg/dL) were confirmed as diabetic and selected for further experiments.

### 2.3. RNA Isolation and Real-Time PCR

Total RNA was extracted from either the sample or cultured cells via the FastPure Cell/Tissue Total RNA Isolation Kit V2 (Vazyme, China). According to the manufacturer’s instructions, approximately 1 μg of RNA was then reverse transcribed to cDNA via the PrimeScriptTM RT Reagent Kit with gDNA Eraser (RR047A, Takara, USA). A real-time qPCR assay was carried out with cDNA using the ChamQ Universal SYBR qPCR Master Mix (Q711-02, Vazyme) via the CFX384 detection system (Bio-Rad, USA), and *Actin* served as an internal reference. The expression data were analyzed via the 2-ΔΔCt method. The primer sequences used for RT-qPCR were as follows: *Npy*, forward 5′-TACTCCGCTCTGCGACACTACA-3′ and reverse 5′-GGCGTTTTCTGTGCTTTCCTTCA-3′; *Adcyap1*, forward 5′-AGTGTCTCCTGTTCACCTGCCG -3′ and reverse 5′-AGTAAAGGGCGTAAGCGTCACG-3′; and *Actin*, forward 5′-CATTGCTGACAGGATGCAGAAGG-3′ and reverse 5′-TGCTGGAAGGTGGACAGTGAGG-3′.

### 2.4. ATAC-Seq Assay

The procedures were conducted according to the protocol [17]. A hypothalamus tissue fragment of ∼100 mg was placed into a pre-chilled 2-mL tube containing 0.6 mL of cold 1× homogenization buffer (320 mM sucrose, 0.1 mM EDTA, 0.1% NP40, 5 mM CaCl_2_, 3 mM Mg(Ac)_2_, 10 mM Tris pH 7.8, 1× protease inhibitors (Complete, Roche, Switzerland), and 167 μM β-mercaptoethanol in water. The tissue was homogenized using a pipette by 20 strokes. The connective tissue and residual debris were precleared by filtration through an 80-μm nylon mesh filter followed by centrifugation for 5 min at 100× *g*. To avoid pelleted debris, 400 μL was transferred to a pre-chilled 2 mL round-bottom Lo-Bind tube (EP022431048, Eppdendorf, USA). In a swinging-bucket centrifuge, the nuclei were centrifuged for 20 min at 3000 r.c.f. using iodixanol. After centrifugation, the nuclei were present at the interface of the 29% and 35% iodixanol solutions.

A total of 50,000 counted nuclei were then transferred to a tube containing 1 mL of ATAC-seq RSB with 0.1% Tween-20. Nuclei were pelleted by centrifugation at 500 r.c.f. for 10 min in a pre-chilled (4 °C) fixed-angle centrifuge. The supernatant was removed via the two pipetting steps described above. The transposition mixture (25 μL of 2 × TD buffer, 2.5 μL of Tn5 (100 nM final), 16.5 μL of PBS, 0.5 μL of 1% digitonin, 0.5 μL of 10% Tween-20, and 5 μL of water) was added directly to the nuclear pellet and mixed by pipetting up and down six times. The samples were incubated at 37 °C for 30 min in a thermomixer with shaking at 1000 r.p.m. Tagmented DNA was collected via tagmentation DNA extraction beads and then amplified via PCR. The DNA libraries were purified via DNA clean beads and then sequenced.

### 2.5. Nuclei Isolation and ATAC-Seq Library Preparation

Hypothalamic nuclear extraction and ATAC-seq library preparation were adapted from previously described protocols [17]. Briefly, freshly harvested mouse hypothalamic tissue was immediately submerged in 0.6 mL of ice-cold 1× homogenization buffer composed of 320 mM sucrose, 10 mM Tris-HCl (pH = 7.8), 5 mM CaCl_2_, 3 mM Mg(Ac)_2_, 0.1 mM EDTA, 0.1% NP-40, 167 μM β-mercaptoethanol, and 1× protease inhibitor cocktail (Complete, Roche, Switzerland). Tissue dissociation was achieved by gentle mechanical trituration (20 strokes using a pipette). The homogenate was filtered through an 80 μm nylon cell strainer to remove connective tissue remnants, followed by low-speed centrifugation at 100× *g* for 5 min to settle large cellular debris. A 400-μL portion of the cleared lysate was transferred to a pre-chilled 2 mL Lo-Bind tube and subjected to density gradient centrifugation at 3000× *g* for 20 min at 4 °C in a swinging-bucket rotor. Intact nuclei were selectively harvested from the 29%/35% iodixanol interface. A total of 50,000 counted nuclei were washed in 1 mL of ATAC-RSB supplemented with 0.1% Tween-20 and pelleted at 500× *g* for 10 min (4 °C, fixed-angle rotor).

Following total removal of the supernatant, the intact nuclear pellet was resuspended in 50 μL of transposition cocktail containing 25 μL 2× TD buffer, 2.5 μL Tn5 transposase (100 nM final concentration), 16.5 μL PBS, 0.5 μL 1% digitonin, 0.5 μL 10% Tween-20, and 5 μL nuclease-free water. Chromatin tagmentation was performed at 37 °C for 30 min under continuous shaking (1000 rpm). Tagmented DNA fragments were captured with DNA extraction beads, PCR-amplified, purified using DNA cleanup beads, and finally subjected to high-throughput sequencing.

### 2.6. Single-Nucleus RNA Sequencing (snRNA-Seq)

HFD/STZ-induced T2D model mice and age-matched non-pregnant controls were transcardially perfused with PBS. Brains were rapidly dissected on an ice-cooled plate, and hypothalamic regions were precisely isolated with reference to a standard mouse brain atlas. Tissues were homogenized to generate high-quality single-nucleus suspensions. Isolated nuclei were partitioned into Gel Beads-in-Emulsion (GEMs) using the 10x Genomics Chromium system to perform nuclear barcoding and full-length cDNA synthesis. Sequencing libraries were constructed according to the manufacturer’s instructions (10x Genomics) and subsequently sequenced on an Illumina high-throughput platform. Raw sequencing datasets underwent stringent quality filtration, alignment, and downstream bioinformatic visualization. After snRNA-seq, the raw datasets in Fastq format of the control and T2D groups were preprocessed and aligned to the mouse reference genome (GRCm38) by Cell Ranger 8.0.1. The preprocessed gene and cell matrix was transformed into a Seurat object and filtered using the Seurat package (v5.5.1), and the cells remained in terms of percent of mitochondria per cell less than 30, UMI numbers between 1000 and 30,000, and gene number smaller than 8000 genes. The doublet situation of these datasets was checked by the DoubletFinder package (v2.0.6), but the number and distribution of doublets in UMAP are sparse and dispersed, so removal is not necessary. Before cell annotation, these two datasets were normalized and integrated to alleviate the batch effect using the RPCA algorithm in the Seurat package. The cell type annotation of this study was achieved in two steps: major cell populations were first identified based on established markers [18,19], followed by refined subclustering of neuronal and oligodendrocyte-lineage populations according to reference annotations and marker genes [20,21,22].

### 2.7. Sequencing QC

The raw reads from ATAC-seq were trimmed and filtered by fastp (v0.23.4) [23] with the following parameters: (-g -q 5 -u 50 -n 15 -l 15 --overlap_diff_limit 1 --overlap_diff_percent_limit 10). Quality control was performed via FastQC (v0.12.1, https://github.com/s-andrews/FastQC, accessed on 3 August 2026) software.

### 2.8. ATAC-Seq Data Analysis

ATAC-seq reads were processed through the ENCODE ATAC-seq standard pipeline (v2.2.3, https://github.com/ENCODE-DCC/atac-seq-pipeline, accessed on 3 August 2026). Clean reads were aligned to GRCh38 or GRCm38 via Bowtie2 with “ -X2000 --mm” and duplicated via Picard (v2.27.5) MarkDuplicates command with “VALIDATION_STRINGENCY = SILENT” parameters. The mapped reads were then shifted +4 bp for the positive strand and -5 bp for the negative strand to correct for Tn5 insertion bias. ATAC peaks were subsequently identified via MACS2 with the “-p 0.01 --shift -75 --extsize 150 --nomodel -B --SPMR --keep-dup all --call-summits” options, and peaks overlapping with ENCODE mm10 blacklist regions were removed via bedtools (v2.31.0) [24]. Genome-wide ATAC-seq single tracks were generated via bamCoverage from deepTools (v3.5.4) with the “-binSize 10” option. Differential accessibility regions (DARs) between the experimental and control groups were analyzed via DiffBind. (v3.10.1) R package with *p* value < 0.05 and |log2Fold-change| >= 1 as the thresholds. All heatmaps were generated via deepTools (v3.5.4), and genomic coverage tracks were visualized via Figeno (v1.8.1) [25].

### 2.9. Single-Nucleus RNA-Sequencing Data and Cell Annotation

Downstream analyses were performed using quality-controlled, integrated, clustered, and annotated single-nucleus RNA-sequencing datasets generated from control and T2D samples. Experimental conditions were obtained from the experiment field in the Seurat metadata. Major cell classes were defined according to the resolu_RNA_0.1_anno annotation, whereas fine-grained cell subtypes were assigned using resolu_overall_aftersub_anno. The major cell populations included neurons, astrocytes, oligodendrocytes and oligodendrocyte precursor cells, microglia, vascular cells, and tanycytes. The major-cell analysis comprised 14,490 nuclei from control samples and 21,396 nuclei from T2D samples. Analyses were conducted in R version 4.5.2 using Seurat version 5.4.0, SeuratObject version 5.2.0, monocle version 2.38.0, CellChat version 2.2.0, clusterProfiler version 4.18.4, org.Mm.eg.db version 3.22.0, ggplot2 version 4.0.2, and patchwork version 1.3.2.

### 2.10. Dimensionality Reduction and Cell-Composition Analysis

Cellular distributions were visualized using the uniform manifold approximation and projection (UMAP) coordinates stored in the annotated Seurat objects. Major cell classes and fine cell subtypes were visualized separately for control and T2D samples. For each condition, the relative abundance of a cell population was calculated as the number of nuclei assigned to that population divided by the total number of nuclei in the corresponding condition. Cell-composition analyses were performed independently at the major-cell-class and fine-subtype levels.

### 2.11. Marker-Gene Expression Analysis

Marker-gene expression was evaluated using normalized expression values from the RNA assay. For each gene and cell population, average expression was calculated after back-transformation of the log-normalized values and subsequent log transformation of the population-level mean. The percentage of expressing nuclei was defined as the proportion of nuclei with a normalized expression value greater than zero. For visualization, average expression values were standardized across cell populations for each gene and restricted to a range of −1 to 2. The major-cell marker panel included markers of excitatory neurons (*Snap25*, *Slc17a7*, *Satb2*, *Col5a1*, and *Slc17a6*), inhibitory neurons (*Gad1*, *Gad2*, and *Slc32a1*), astrocytes (*Aldh1l1*, *Gfap*, *Slc1a3*, and *Slc1a2*), microglia (*Tmem119*, *Hexb*, *C1qa*, *Cx3cr1*, *Ly86*, *P2ry12*, and *Itgam*), oligodendrocytes (*Plp1*, *Mbp*, *Cldn11*, *Mog*, *Olig2*, and *Mag*), oligodendrocyte precursor cells (OPCs; *Pdgfra*, *Vcan*, *Cspg4*, and *Olig1*), and endothelial cells (*Vtn*, *Flt1*, *Vwf*, and *Cldn5*).

Expression patterns across the oligodendrocyte lineage were additionally examined in OPCs, newly formed oligodendrocytes, myelinating oligodendrocytes, and *Cntnap2*^+^ oligodendrocytes. The lineage marker panel included *Pdgfra*, *Cspg4*, *Vcan*, *Sox6*, *Gpr17*, *Tcf7l2*, *Bcas1*, *Enpp6*, *Myrf*, *Mbp*, *Plp1*, *Mog*, *Mag*, *Mobp*, *Cnp*, *Sox10*, *Olig1*, *Olig2*, *Cntnap2*, *Cntn5*, and *Galntl6*.

### 2.12. Differential Expression and Functional Enrichment Analyses

Differential expression between T2D and control samples was assessed independently within each major cell class. Only cell classes containing at least 20 nuclei in each condition were included. Differentially expressed genes were identified using the Wilcoxon rank-sum test implemented in Seurat. Genes expressed in at least 5% of nuclei in either comparison group were tested without applying an initial fold-change threshold. Statistical significance was defined as a Benjamini–Hochberg-adjusted *p* value < 0.05 and an absolute average log_2_ fold change ≥ 0.25. Positive fold-change values indicated higher expression in T2D, whereas negative values indicated higher expression in the control.

Gene Ontology biological process enrichment analysis was performed using the enrichGO function in clusterProfiler. Mouse gene symbols were converted to Entrez Gene identifiers using org.Mm.eg.db. Enrichment was evaluated using the biological process ontology, and multiple-testing correction was performed using the Benjamini–Hochberg method. All genes detected in the RNA assay and successfully mapped to Entrez identifiers were used as the background gene universe. For visualization, the six most significantly enriched biological processes were selected for each major cell class.

### 2.13. Analysis of Selected Neuronal Subtypes

Three neuronal subtypes—Gal.Nts.Npy.Other, Nxph4.Adcyap1.GLU, and Lmx1a.Gpr149.Tcf4.GLU—were selected for detailed analysis. The numbers of the control and T2D nuclei were 726 and 9624 for Gal.Nts.Npy.Other, 126 and 1508 for Nxph4.Adcyap1.GLU, and 77 and 536 for Lmx1a.Gpr149.Tcf4.GLU.

Within each neuronal subtype, differential expression between T2D and control was assessed using the Wilcoxon rank-sum test. Genes expressed in at least 5% of nuclei were included, and significant differential expression was defined as an adjusted *p* value < 0.05 and an absolute average log_2_ fold change ≥ 0.25. Functional enrichment was performed separately for each neuronal subtype. The background universe consisted of all genes tested in the corresponding differential expression analysis that could be mapped to Entrez identifiers. The five highest-ranking biological processes were retained for visualization. Expression distributions of genes associated with metabolic signaling, neuropeptide activity, and immediate-early responses were further examined, including *Lepr*, *Insr*, *Socs3*, *Ptpn1*, *Npy*, *Gal*, *Nts*, *Adcyap1*, *Adcyap1r1*, *Fos*, *Jun*, and *Egr1*.

### 2.14. Oligodendrocyte-Lineage Trajectory Analysis

Trajectory analysis was performed on nuclei annotated as OPCs, newly formed oligodendrocytes, myelinating oligodendrocytes, or *Cntnap2*^+^ oligodendrocytes. The final trajectory dataset contained 2687 control nuclei and 302 T2D nuclei. A Monocle2 CellDataSet was constructed from RNA count data using a negative binomial expression model. Size factors and dispersion parameters were estimated before gene detection. Genes expressed in at least 20 nuclei were retained for trajectory inference. Ordering genes were selected based on their association with oligodendrocyte-lineage stages. Genes with a false-discovery-rate-adjusted q value < 1 × 10^−5^ were prioritized. When fewer genes met this criterion, the most significant genes were selected, with the final ordering gene set restricted to a maximum of 1500 genes.

Dimensionality reduction was performed using the DDRTree algorithm, followed by pseudotemporal ordering of nuclei. The trajectory root was assigned to the state containing the highest proportion of OPCs, corresponding to state 3. Differences in pseudotime distributions between the control and T2D were evaluated using Wilcoxon rank-sum tests. For comparisons performed within individual lineage stages, *p* values were adjusted using the Benjamini–Hochberg method. Genes associated with pseudotime were identified using spline-based differential expression models with three degrees of freedom.

Branch-dependent expression patterns were assessed using branched expression analysis modeling at branch point 1. Genes with a q-value < 1 × 10^−5^ were considered branch-associated, and the 60 most significant genes were visualized in a hierarchically clustered heatmap. Branched expression trends of the myelination-related genes *Mbp*, *Mobp*, *Mog,* and *Myrf* were examined along pseudotime.

### 2.15. OPC Gene-Module Scoring

Gene-module scores were calculated within the OPC population using Seurat’s AddModuleScore function. Three biologically defined gene sets were evaluated. The proliferation module comprised *Mki67*, *Top2a*, *Pcna*, *Cdk1*, *Ccna2*, *Ccnb1*, *Ube2c*, *Mcm2*, *Mcm3*, *Mcm4*, *Mcm5*, and *Mcm6*. The myelination module included *Myrf*, *Mbp*, *Plp1*, *Mog*, *Mag*, *Mobp*, *Cnp*, and *Opalin.* The *Cntnap2*-associated state module included *Cntnap2*, *Cntn5*, *Galntl6*, *Nrg1*, *Nrg3*, *Lingo2*, *Kcnip4*, *Tenm1*, *Tenm2*, and *Lrfn5*. Module scores were calculated using 24 expression bins and 100 control genes per analyzed feature. Differences in module scores between Control and T2D OPCs were assessed using Wilcoxon rank-sum tests.

### 2.16. Cell–Cell Communication Analysis

Cell–cell communication networks were inferred separately for Control and T2D samples using CellChat and the CellChatDB.mouse ligand–receptor database. The analysis included neurons, astrocytes, microglia, OPCs, and *Cntnap2*^+^ oligodendrocytes. Cell groups were defined by combining the major cell-class and fine-subtype annotations. To reduce the influence of the substantially larger neuronal population, neurons were randomly downsampled to a maximum of 4000 nuclei per condition, whereas all available nuclei from the other selected populations were retained. A fixed random seed was used to ensure reproducibility.

Communication probabilities were estimated from normalized RNA expression using the truncated-mean approach. Population-size weighting and spatial-distance constraints were not applied. Ligand–receptor interactions involving cell groups represented by fewer than 10 nuclei were excluded. Communication probabilities were estimated using 80 bootstrap iterations and subsequently summarized at the signaling-pathway level.

Condition-specific CellChat objects were merged to compare the overall number and strength of predicted interactions between control and T2D. Pairwise communication strength was defined as the sum of significant ligand–receptor communication probabilities for each source–target cell pair. Differential communication was calculated as the T2D value minus the corresponding control value. For pathway-level visualization, the 12 pathways with the highest total communication probability were selected, and communication strengths were displayed after log_1+_ transformation.

### 2.17. Pathway Activity Scores Analysis

Pathway activity scores were calculated using the AddModuleScore function in Seurat based on log-normalized RNA expression. Mouse Gene Ontology biological process gene sets were intersected with genes detected in the snRNA-seq dataset, and gene sets containing 10–500 detected genes were retained. For each cell and pathway, the mean expression of pathway genes was calculated and subtracted from the mean expression of 100 expression-matched control genes per feature bin (ctrl = 100, nbin = 24). Module scores were subsequently averaged within each oligodendrocyte subtype and condition.

## 3. Results

### 3.1. T2D Induces Marked Remodeling of Hypothalamic Cellular Composition Revealed by Single-Nucleus RNA Sequencing

**Figure 1:**
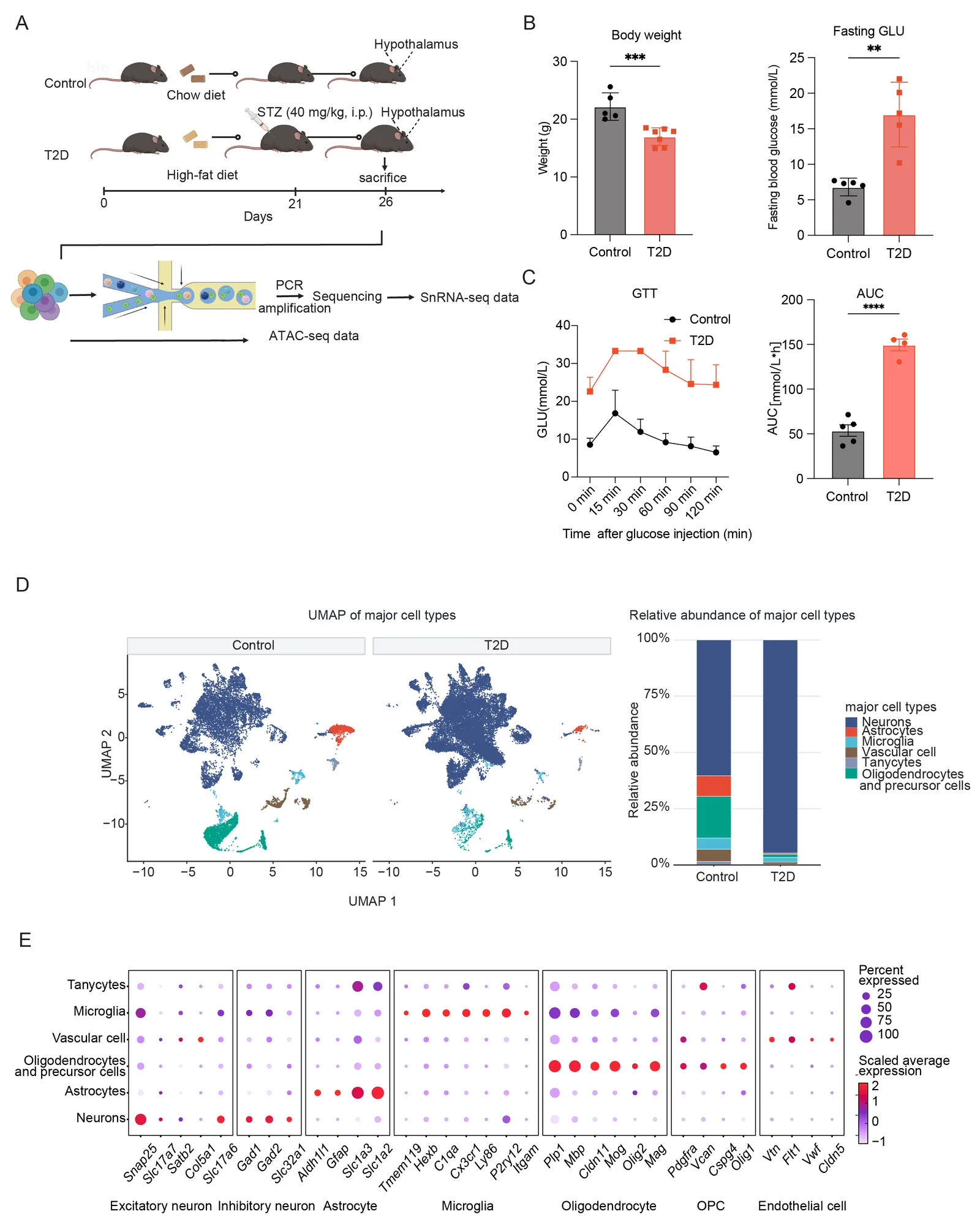
Metabolic characterization and single-nucleus profiling of the hypothalamus in HFD/STZ-treated T2D mice. (**A**) Experimental workflow. Female mice were fed either a chow diet (control) or a high-fat diet for 3 weeks. HFD-fed mice subsequently received four consecutive daily STZ injections (40 mg/kg, i.p.). Hypothalamic tissues were collected after STZ administration for single-nucleus RNA sequencing and ATAC-seq. For snRNA-seq, hypothalamic tissues from three mice within each condition were pooled before nuclei isolation and library preparation. (**B**) Metabolic characteristics of control and T2D mice before tissue collection. Body weight and fasting blood glucose levels are shown. Data are presented as mean ± SEM. Statistical significance was determined using an unpaired two-tailed Student’s *t*-test. ** *p* < 0.01, *** *p* < 0.001. (**C**) Glucose tolerance test (GTT) curves and corresponding area under the curve (AUC) in control and T2D mice (*n* = 5 and 4, respectively). Data are presented as mean ± SEM. Statistical significance for AUC was determined using an unpaired two-tailed Student’s *t*-test. **** *p* < 0.0001. (**D**) UMAP visualization of major hypothalamic cell types identified by snRNA-seq in the pooled control and T2D datasets. Stacked bar plots show the relative abundance of major cell populations in each condition. (**E**) Canonical marker gene expression used for annotation of major hypothalamic cell populations, including excitatory neurons, inhibitory neurons, astrocytes, microglia, oligodendrocytes, oligodendrocyte precursor cells (OPCs), endothelial cells, vascular cells, and tanycytes.

To investigate the cellular response of the hypothalamus to type 2 diabetes (T2D), we established a mouse model by combining three weeks of 60 kal% high-fat diet (HFD) feeding with four consecutive daily intraperitoneal injections of streptozotocin (STZ, 40 mg/kg) [26,27]. Hypothalamic tissue was subsequently collected for single-nucleus RNA sequencing (snRNA-seq) and bulk ATAC-seq (Figure 1A). Compared with control mice, T2D mice exhibited significantly lower body weight and markedly elevated fasting blood glucose levels (Figure 1B). Glucose tolerance testing (GTT) further revealed substantially impaired glucose clearance, with persistently elevated blood glucose concentrations following glucose administration and a significantly increased area under the curve (AUC) compared with controls (Figure 1C). Together, these metabolic phenotypes demonstrate that HFD/STZ treatment produced a robust diabetic state characterized by fasting hyperglycemia and pronounced glucose intolerance.

Following quality control, sample integration, and unsupervised clustering, six major hypothalamic cell populations were identified: neurons, astrocytes, oligodendrocytes and oligodendrocyte precursor cells (OPCs), microglia, vascular cells, and tanycytes (Figure 1D). Cell identities were assigned according to established canonical marker genes [18,19,22,28,29] (Figure 1E and Figure S1). Excitatory neurons were characterized by expression of *Slc17a7* and *Slc17a6*, whereas inhibitory neurons expressed *Gad1* and *Gad2*. Astrocytes were identified by *Slc1a2*, *Slc1a3*, and *Gfap*; oligodendrocyte-lineage cells by *Plp1*, *Mbp*, *Mog*, and *Mag*; and OPCs by *Pdgfra*, *Cspg4*, and *Sox10*. Microglia expressed *Cx3cr1* and *P2ry12*, vascular cells expressed *Flt1*, *Vwf*, and *Cldn5*, and tanycytes expressed *Rax* and *Col25a1*. Comparison of cell composition between the two groups indicated substantial remodeling of the hypothalamic cellular landscape in T2D. Notably, neuronal populations occupied a substantially greater proportion of total cells in T2D mice, whereas the relative representation of astrocytes and oligodendrocyte-lineage cells was reduced (Figure 1D). Because snRNA-seq measures relative cellular abundance rather than absolute cell numbers, these differences should be interpreted as disease-associated shifts in cellular representation. These findings demonstrate that T2D is accompanied by broad changes in the cellular organization of the hypothalamus, providing a basis for subsequent analyses of neuronal and glial remodeling.

### 3.2. HFD/STZ-Induced Diabetic Conditions Are Associated with Cell Type-Specific Transcriptional Remodeling in the Hypothalamus

**Figure 2:**
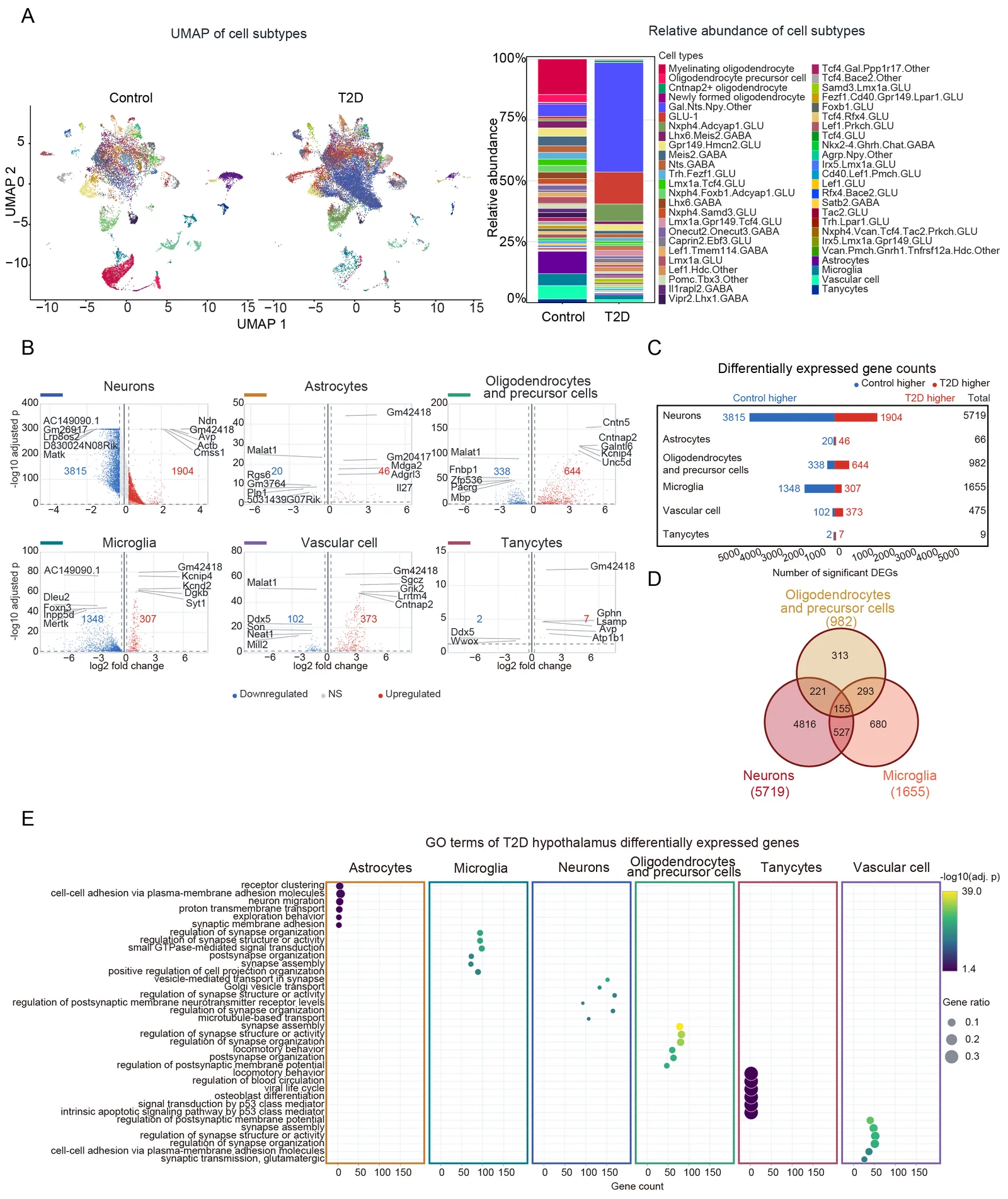
HFD/STZ-induced diabetic conditions are associated with remodeling of hypothalamic cellular composition and cell type–specific transcriptional programs. (**A**) UMAP visualization of annotated hypothalamic cell subtypes from control and T2D mice, together with the relative abundance of each subtype in the two conditions. (**B**) Volcano plots showing differentially expressed genes (DEGs) identified in each major cell type between control and T2D mice (adjusted *p* < 0.05). (**C**) Number of differentially expressed genes detected in each major cell type. Neurons exhibited the largest number of DEGs, followed by microglia and oligodendrocyte-lineage cells. (**D**) Venn diagram illustrating shared and cell type-specific DEGs among neurons, microglia, and oligodendrocyte-lineage cells. Although most DEGs were cell type-specific, a subset of genes was shared across these three cell populations. (**E**) Gene Ontology enrichment analysis of DEGs identified in individual major cell types, highlighting distinct functional programs associated with T2D.

To determine which hypothalamic cell populations were preferentially affected under HFD/STZ-induced diabetic conditions, we performed higher-resolution sub-clustering and identified multiple transcriptionally distinct neuronal and non-neuronal subtypes (Figure 2A and Figure S2). Comparison of subtype composition analysis revealed prominent alterations in several neuronal populations, including Gal.Nts. Npy neurons, Nxph4.Adcyap1 glutamatergic neurons and Lmx1a.Gpr149.Tcf4 glutamatergic neurons, together with changes in oligodendrocyte-lineage populations. These observations suggest that diabetic conditions are astypes (Figure 2A).

Next, we performed cell type-specific differential expression analysis to characterize transcriptional responses associated with diabetic conditions. Neurons exhibited the most extensive transcriptional remodeling, yielding 5719 differentially expressed genes (DEGs), followed by microglia (1655 DEGs) and oligodendrocyte-lineage cells (982 DEGs). In contrast, astrocytes, vascular cells, and tanycytes displayed comparatively modest transcriptional shifts (Figure 2B,C). These results indicate that transcriptional responses under diabetic conditions are highly heterogeneous across hypothalamic cell types, with particularly extensive remodeling in neurons and substantial molecular alterations in microglial and oligodendrocyte-lineage populations.

Although the majority of DEGs were cell type-specific, intersection analysis identified a core subset of genes shared among neurons, microglia, and oligodendrocyte-lineage cells (Figure 2D), suggesting that these populations engage both common and cell type-specific transcriptional responses under diabetic conditions. Gene Ontology enrichment analysis further highlighted distinct functional programs associated with these responses (Figure 2E). Neuronal DEGs were predominantly enriched in processes related to synaptic organization, synaptic signaling, neurotransmitter transport, and regulation of postsynaptic membrane potential, consistent with broad alterations in neuronal communication. In oligodendrocyte-lineage cells, DEGs were enriched in pathways associated with myelination, axon ensheathment, and oligodendrocyte differentiation, indicating perturbation of oligodendrocyte maturation and myelin-related programs. Microglial DEGs were mainly associated with cytoskeletal organization and small GTPase-mediated signaling, whereas vascular cell DEGs were enriched in processes related to cell adhesion and vascular remodeling (Figure 2E).

Collectively, these findings demonstrate cell type-specific transcriptional remodeling in the hypothalamus under diabetic conditions. In particular, the prominent remodeling of neuronal subtypes, together with alterations in oligodendrocyte differentiation and myelination-associated programs, prompted us to further investigate neuronal subtype-specific responses and oligodendrocyte-lineage state transitions in subsequent analyses.

### 3.3. T2D Is Associated with Widespread Remodeling of Hypothalamic Chromatin Accessibility

**Figure 3:**
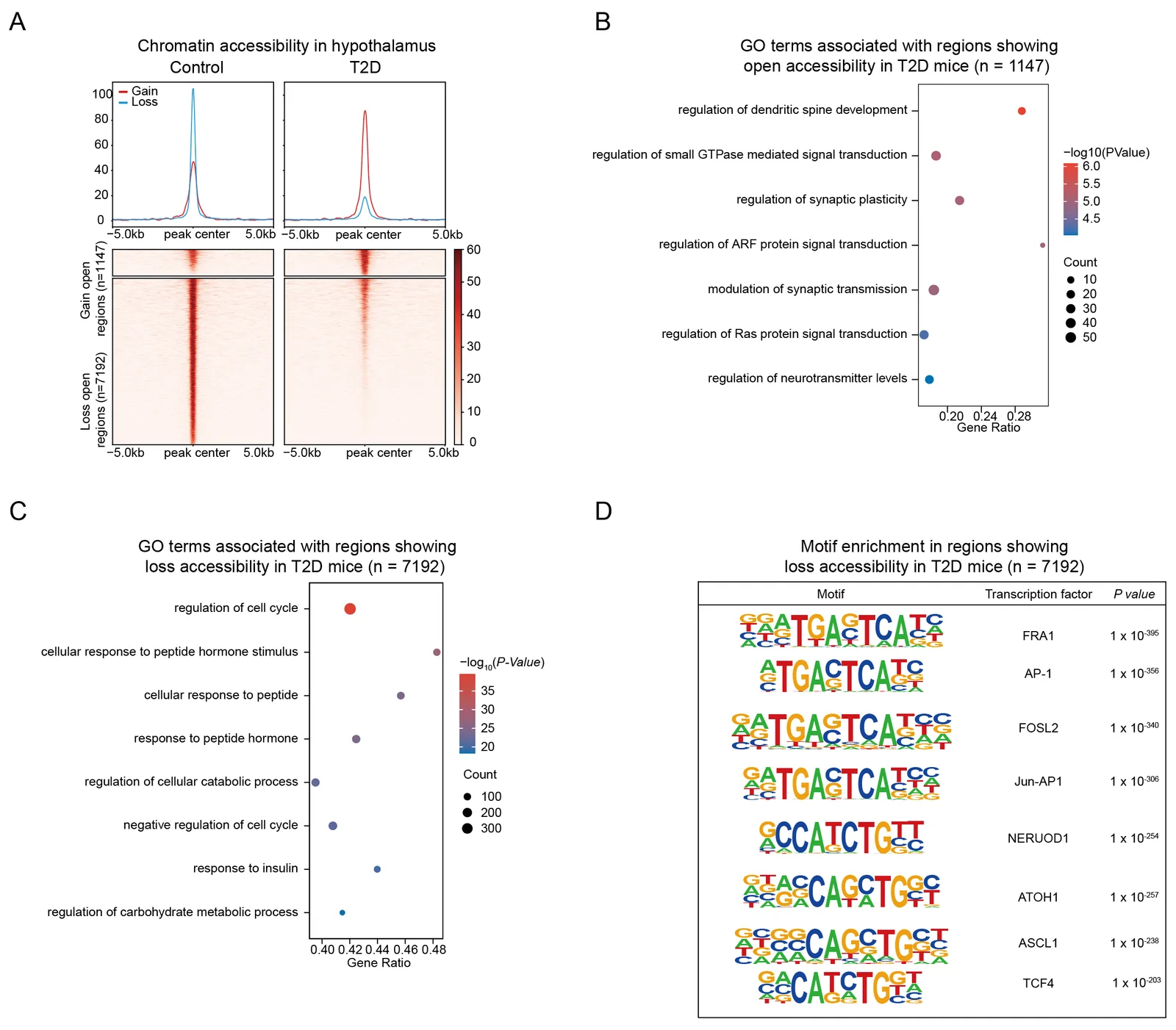
Chromatin accessibility differences in the hypothalamus of T2D mice. (**A**) Aggregate ATAC-seq signal profiles and heatmaps centered on regions showing altered chromatin accessibility between pooled control and T2D hypothalamic samples. Hypothalamic tissues from three mice within each condition were pooled before ATAC-seq library preparation, resulting in one pooled library per condition. Regions with increased accessibility (*n* = 1147) and decreased accessibility (*n* = 7192) in T2D mice relative to controls are shown. ATAC-seq signals are displayed within ±5 kb of the peak center. (**B**) Gene Ontology (GO) enrichment analysis of genes associated with regions showing increased chromatin accessibility in T2D datasets. (**C**) Gene Ontology enrichment analysis of genes associated with regions showing decreased chromatin accessibility in T2D datasets. (**D**) Transcription factor motif enrichment analysis of regions showing decreased chromatin accessibility in T2D datasets.

To investigate whether cellular and transcriptomic alterations observed in T2D mice were accompanied by changes in the hypothalamic regulatory landscape, we performed a bulk assay for transposase-accessible chromatin using sequencing (ATAC-seq) on hypothalamic tissues from control and T2D mice. Genome-wide chromatin accessibility profiling revealed extensive differences between the two groups (Figure 3A). Aggregate ATAC-seq signal profiles and heatmap analysis within 5 kb of peak centers identified thousands of differentially accessible regions (DARs). Specifically, T2D led to a prominent loss of chromatin accessibility across 7192 regions, alongside a subset of 1147 regions exhibiting gained accessibility relative to controls (Figure 3A). Thus, the overall accessibility landscape was characterized by a predominance of accessibility loss together with a smaller subset of regions showing increased accessibility. Because ATAC-seq measures chromatin accessibility rather than specific DNA or histone modifications, the molecular mechanisms underlying these changes cannot be resolved from the present data alone [30,31,32].

We performed Gene Ontology (GO) enrichment analysis of genes associated with DARs to uncover the biological pathways linked to these accessibility alterations. Regions showing increased accessibility in T2D mice were associated with processes related to neuronal connectivity and synaptic function, including regulation of ion transport, modulation of synaptic transmission, regulation of synaptic plasticity, vesicle-mediated transport, and regulation of neuron projection development (Figure 3B). These data suggest that increased chromatin accessibility is preferentially associated with regulatory programs related to neuronal and synaptic function. In contrast, regions displaying decreased chromatin accessibility were associated with processes including regulation of cell cycle, cellular response to peptide hormone stimulus, response to peptide, response to insulin, regulation of cellular catabolic processes, and regulation of carbohydrate metabolic processes (Figure 3C). This pattern suggests that diabetic stress is associated with broad changes in chromatin accessibility at genomic regions linked to neuronal signaling, hormone responsiveness, and metabolic regulation.

To further characterize the regulatory features of these DARs, we performed transcription factor (TF) motif enrichment analysis. Regions with decreased accessibility were enriched for motifs of AP-1 family members including FRA1, AP-1, FOSL2, Jun-AP1, as well as motifs corresponding to neurodevelopmental and basic helix-loop-helix transcription factors such as NEUROD1, ATOH1, ASCL1, and TCF4 (Figure 3D). In contrast, gained open chromatin regions showed significant enrichment for Regulatory Factor X (RFX) family motifs (RFX, RFX2, X-box) [33,34] and specific AP-1 subcomponents (FRA2, FOS, FRA1, JUNB; Figure S3A). These motif-enrichment patterns suggest that distinct transcription factor-associated regulatory programs may contribute to the chromatin accessibility differences observed between the control and T2D hypothalamus. However, motif enrichment alone does not establish transcription factor activity or direct regulatory control.

To illustrate locus-specific accessibility changes, we inspected individual neuroendocrine gene loci. A region near the *Avp* (arginine vasopressin) locus showed increased ATAC-seq signal in T2D mice compared to controls (Figure S3B). Given the established role of the *Avp* regulatory locus in the activation of stress-regulatory and osmoregulatory neuroendocrine circuits [35,36,37] in diabetes. Taken together, these findings demonstrate that T2D is associated with widespread remodeling of hypothalamic chromatin accessibility, characterized by extensive accessibility loss together with more selective accessibility gains at regions linked to neuronal, synaptic, metabolic, and neuroendocrine regulatory programs.

### 3.4. Condition-Associated Transcriptional Alterations in Selected Hypothalamic Neuronal Populations and Concordant Npy Changes in T2D Mice

**Figure 4:**
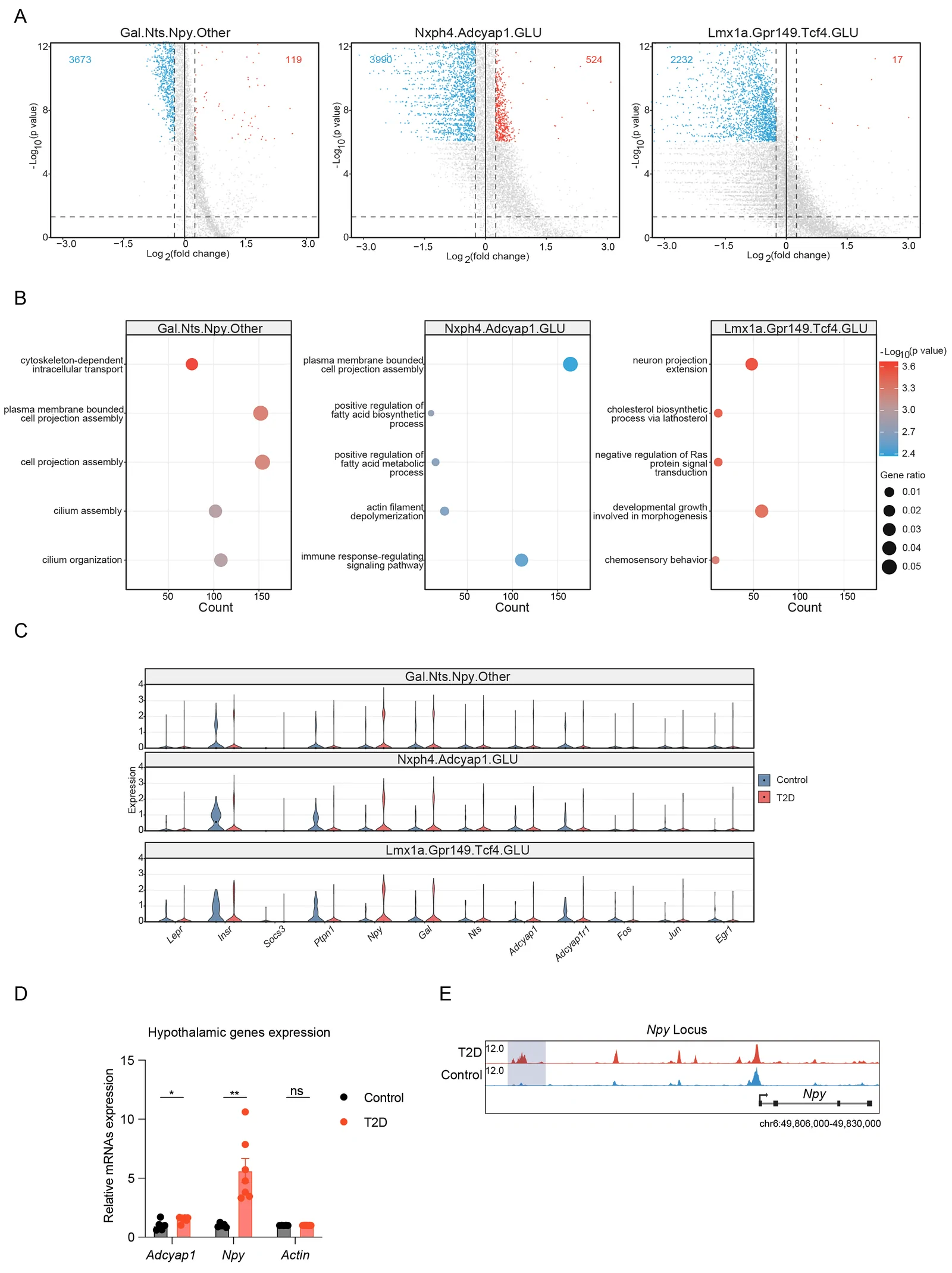
Integrated analyses highlight *Npy*-associated transcriptional and chromatin-accessibility alterations in the T2D hypothalamus. (**A**) Volcano plots showing cell-level differential-expression screening between the T2D and control snRNA-seq datasets with Gal.Nts.Npy, Nxph4.Adcyap1 GLU, and Lmx1a.Gpr149.Tcf4 GLU neurons. Red and blue dots indicate significantly upregulated and downregulated genes in T2D, respectively. (**B**) Gene Ontology (GO) enrichment analysis of condition-associated genes identified within the three selected neuronal populations. Dot size represents the gene ratio/count as indicated, and color represents enrichment significance. Gal.Nts. Npy neurons were enriched for intracellular transport and cell projection organization; Nxph4.Adcyap1 GLU neurons showed enrichment of lipid metabolic and immune-related signaling pathways; Lmx1a.Gpr149.Tcf4 GLU neurons were enriched for neuronal projection development and cholesterol biosynthetic processes. (**C**) Violin plots showing expression of representative genes associated with metabolic sensing, neuropeptide signaling, and activity-related responses in the pooled Control and T2D datasets, including *Lepr*, *Insr*, *Socs3*, *Ptpn1*, *Npy*, *Gal*, *Nts*, *Adcyap1*, *Adcyap1r1*, *Fos*, *Jun*, and *Egr1*. (**D**) Relative hypothalamic mRNA expression of *Adcyap1* and *Npy* in control (*n* = 5) and T2D mice (*n* = 7), measured by RT-qPCR. Data are presented as mean ± SEM. Statistical significance was determined using an unpaired two-tailed Student’s t-test. * *p* < 0.05, ** *p* < 0.01; ns, not significant. (**E**) Integrative genomics viewer snapshot showing ATAC-seq signal distribution at the *Npy* gene loci. T2D and control accessibility signals are shown in red and blue, respectively. The shaded region highlights a genomic interval exhibiting greater accessibility in the T2D datasets relative to the control datasets. Gene structure and genomic coordinates are displayed below the accessibility tracks.

To further characterize neuronal populations showing prominent differences in relative representation between the pooled control and T2D snRNA-seq datasets, we focused on three neuronal subtypes: Gal.Nts.Npy, Nxph4.Adcyap1 GLU, and Lmx1a.Gpr149.Tcf4 GLU neurons. Cell-level differential-expression screening identified extensive condition-associated transcriptional differences within all three populations (Figure 4A). A total of 3792 genes met the predefined differential-expression criteria in Gal.Nts.Npy.Other neurons, 4514 in Nxph4.Adcyap1.GLU neurons, and 2249 in Lmx1a.Gpr149.Tcf4.GLU neurons (Figure S4A).

Comparison of the condition-associated gene sets revealed both shared and subtype-specific molecular responses. A total of 1036 genes were commonly altered across all three neuronal populations, whereas each subtype also exhibited a substantial set of uniquely altered genes (Figure S4B). Gene Ontology enrichment analysis further suggested distinct functional characteristics among these transcriptional signatures (Figure 4B). In Gal.Nts.Npy.Other neurons, enriched terms were primarily related to cytoskeleton organization, intracellular transport, and cell projection assembly, consistent with remodeling of neuronal structure and intracellular trafficking. Nxph4.Adcyap1 GLU neurons showed enrichment of terms related to cell-projection organization, fatty acid metabolic processes, actin filament organization, and immune response-regulating signaling pathways, suggesting coordinated alterations in neuronal projection organization, lipid metabolism, and neuroimmune-related signaling. In Lmx1a.Gpr149.Tcf4 GLU neurons, enriched terms included neuron-projection extension, cholesterol biosynthesis, and Ras protein signal transduction, suggesting changes in neuronal plasticity and membrane-associated metabolic programs under diabetic conditions. Together, these analyses suggest heterogeneous transcriptional responses among selected hypothalamic neuronal states in the pooled T2D dataset.

We next evaluated the genes involved in metabolic sensing, neuropeptide signaling, and activity-associated responses across these neuronal populations (Figure 4C). Single-nucleus transcriptomics revealed subtype-dependent alterations in the metabolic receptors (*Lepr* and *Insr*) as well as immediate-early genes (*Fos*, *Jun*, and *Egr1*). Among the neuropeptide-associated genes examined, *Npy* showed higher expression in T2D datasets, particular within Gal.Nts.Npy neurons, whereas increased *Adcyap1* expression was observed in Nxph4.Adcyap1 glutamatergic neurons (Figure 4C). These patterns highlight condition-associated alterations in neuropeptide-related transcriptional programs within selected hypothalamic neuronal populations.

We next assessed whether the changes in *Npy* and *Adcyap1* detected in the pooled snRNA-seq dataset were also evident at the whole-hypothalamus level. RT-qPCR analysis of hypothalamic tissues confirmed significantly increased expression of *Adcyap1* and *Npy* in T2D mice (Figure 4D), providing animal-level experimental support for the corresponding transcriptomic observations. In particular, *Npy* showed a marked increase that was consistent with the single-nucleus transcriptomic findings (Figure 4D). To determine whether altered *Npy* expression coincided with changes in the local chromatin-accessibility landscape, we examined bulk ATAC-seq profiles surrounding the *Npy* locus. The T2D ATAC-seq dataset showed greater accessibility within a genomic interval near *Npy* compared with the control dataset (Figure 4E). Thus, increased *Npy* expression was accompanied by greater local chromatin accessibility in the T2D hypothalamus. However, because the ATAC-seq libraries were generated from pooled whole-hypothalamic tissues, this accessibility difference cannot be assigned specifically to Gal.Nts.Npy.Other neurons, and may reflect differences in cellular composition and/or cell-intrinsic chromatin accessibility.

Together, these complementary analyses identify *Npy* as a prominent candidate showing concordant transcriptional and chromatin-accessibility alterations in the T2D hypothalamus. The present data establish an association between increased *Npy* expression and greater accessibility near the *Npy* locus but do not establish a cell-specific epigenetic regulatory mechanism.

### 3.5. T2D Treatment Is Associated with Altered Oligodendrocyte-Lineage Transcriptional States and Increased Representation of a Cntnap2-Positive Population

**Figure 5:**
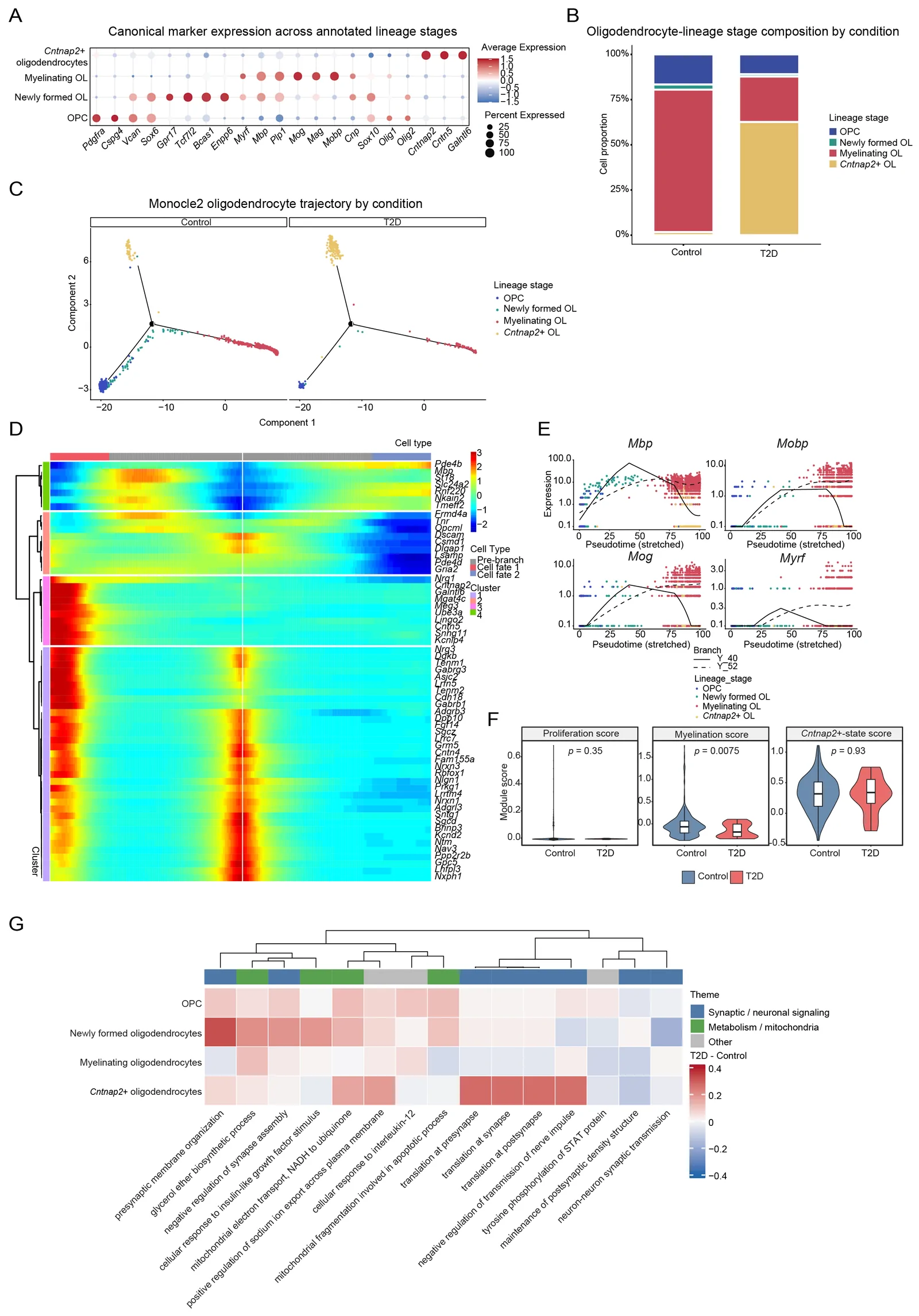
Altered oligodendrocyte-lineage transcriptional states in the T2D hypothalamus. (**A**) Dot plot showing canonical marker expression across annotated oligodendrocyte-lineage stages, including OPCs, newly formed oligodendrocytes, myelinating oligodendrocytes, and *Cntnap2*-positive oligodendrocytes. Dot size represents the percentage of cells expressing each gene, and color indicates scaled average expression. (**B**) Relative representation of oligodendrocyte-lineage transcriptional states within the control and T2D snRNA-seq datasets from mice. The T2D datasets showed greater relative representation of the *Cntnap2*-positive oligodendrocyte population and a reduced representation of myelinating oligodendrocytes. (**C**) Monocle2 pseudotime trajectory of oligodendrocyte-lineage cells separated by condition. Cells were colored according to annotated lineage stage. The inferred trajectory was rooted in the OPC-enriched state. (**D**) BEAM analysis of branch-associated genes around the major Monocle2 branch point. The heatmap illustrates distinct expression dynamics along the two inferred transcriptional branches. Myelin-associated and *Cntnap2*-associated genes showed different branch-related expression patterns. (**E**) Pseudotime-associated expression patterns of representative myelination-associated genes, including *Mbp*, *Mobp*, *Mog*, and *Myrf*, along the two inferred Monocle2 branches. Solid and dashed lines indicate branch-associated fitted expression trends. (**F**) Violin plots showing proliferation-associated, myelination-associated, and *Cntnap2*-associated gene module scores in oligodendrocyte-lineage cells from control and T2D datasets. Module scores are shown as descriptive transcriptional signatures and are not interpreted as animal-level statistical comparisons. (**G**) Heatmap showing differences in pathway-associated scores between T2D and control datasets across four oligodendrocyte-lineage transcriptional states. Rows display the four identified oligodendrocyte-lineage states, and columns represent specific Gene Ontology (GO) biological pathways categorized by functional themes (top annotation bar: metabolism/mitochondria [green], synaptic/neuronal signaling [blue], and other [grey]). The color gradient reflects the score difference between T2D and control conditions (T2D−Control), with red indicating pathway enrichment/upregulation in T2D and blue indicating pathway suppression/downregulation.

Given the prominent transcriptional differences observed within oligodendrocyte-lineage cells, we next examined their transcriptional-state organization in the control and T2D snRNA-seq datasets. Based on canonical marker expression, oligodendrocyte-lineage cells were classified into four major states: OPCs, newly formed oligodendrocytes, myelinating oligodendrocytes, and a *Cntnap2*-positive oligodendrocyte population (Figure 5A). OPCs were characterized by expression of *Pdgfra*, *Cspg4*, *Vcan*, and *Sox6*, whereas newly formed oligodendrocytes expressed higher levels of *Gpr17*, *Tcf7l2*, *Bcas1*, and *Enpp6*. Myelinating oligodendrocytes showed strong expression of mature myelin-associated genes, including *Myrf*, *Mbp*, *Plp1*, *Mog*, *Mag*, *Mobp*, and *Cnp*. In contrast, the *Cntnap2*-positive population showed comparatively weak expression of conventional OPC and mature myelinating markers while expressing *Cntnap2* and a distinct set of associated genes, suggesting a transcriptionally distinct lineage state within the oligodendrocyte-lineage dataset. Because this population is defined primarily from transcriptomic annotation, its cellular identity requires independent validation.

Comparison of cell-state composition between the control and T2D datasets revealed a substantially greater relative representation of the *Cntnap2*-positive population in T2D samples, accompanied by reduced representation of newly formed and myelinating oligodendrocytes (Figure 5B). We next used Monocle2 to examine transcriptional-state relationships among oligodendrocyte-lineage nuclei. The inferred trajectory was rooted in an OPC-enriched state and extended through transcriptional states associated with newly formed oligodendrocytes toward branches characterized by either myelination-associated or *Cntnap2*-associated expression patterns (Figure 5C). Branch-dependent gene expression analysis using BEAM further revealed distinct transcriptional programs along the two inferred branches (Figure 5D). Myelination-associated genes, including *Mbp*, *St18*, *Slc24a2*, and *Nkain2*, showed stronger expression along the myelination-associated branch, whereas the *Cntnap2*-associated branch was characterized by expression of *Cntnap2*, *Cntn5*, *Galntl6*, *Nrg1*, *Nrg3*, and *Lingo2*. Consistently, representative myelin-related genes, including *Mbp*, *Mobp*, *Mog*, and *Myrf*, exhibited distinct expression dynamics across the inferred branches (Figure 5E). These branch-associated patterns describe transcriptional divergence within the dataset but should not be interpreted as evidence of distinct developmental fates.

Gene-module scoring was subsequently used to examine proliferation-associated, myelination-associated, and *Cntnap2*-associated transcriptional programs (Figure 5F). No clear increase in the proliferation-associated signature was observed in the pooled T2D dataset, whereas the myelination-associated score was lower. The *Cntnap2*-associated module did not show a marked global increase across all oligodendrocyte-lineage nuclei, indicating that the increased representation of the *Cntnap2*-positive population is better interpreted as a change in cell-state composition rather than broad induction of the *Cntnap2*-associated program across the entire lineage. Importantly, these transcriptional signatures do not directly measure OPC proliferation, differentiation rates, or lineage transitions in vivo.

Finally, pathway-score analysis revealed state-dependent differences between the pooled control and T2D datasets (Figure 5G). Newly formed oligodendrocytes showed relatively broad changes in pathways related to metabolic and mitochondrial functions, synaptic or neuronal signaling, and cellular organization, whereas myelinating and *Cntnap2*-positive populations showed more selective pathway-associated differences. Together, these analyses indicate that the T2D hypothalamus is associated with an altered distribution of oligodendrocyte-lineage transcriptional states, characterized by increased representation of a *Cntnap2*-positive population and reduced representation of myelination-associated states. These findings support altered oligodendrocyte-lineage homeostasis but do not establish developmental lineage redirection.

### 3.6. HFD/STZ-Induced Diabetic Conditions Are Associated with Altered Predicted Cell–Cell Communication in the Hypothalamus

Having observed an altered distribution of oligodendrocyte-lineage transcriptional states under HFD/STZ-induced diabetic conditions, we next investigated whether these changes were accompanied by alterations in predicted intercellular signaling within the hypothalamus. We therefore inferred ligand–receptor-mediated communication among neurons, astrocytes, microglia, OPCs, and *Cntnap2*-positive oligodendrocyte-lineage cells.

**Figure 6:**
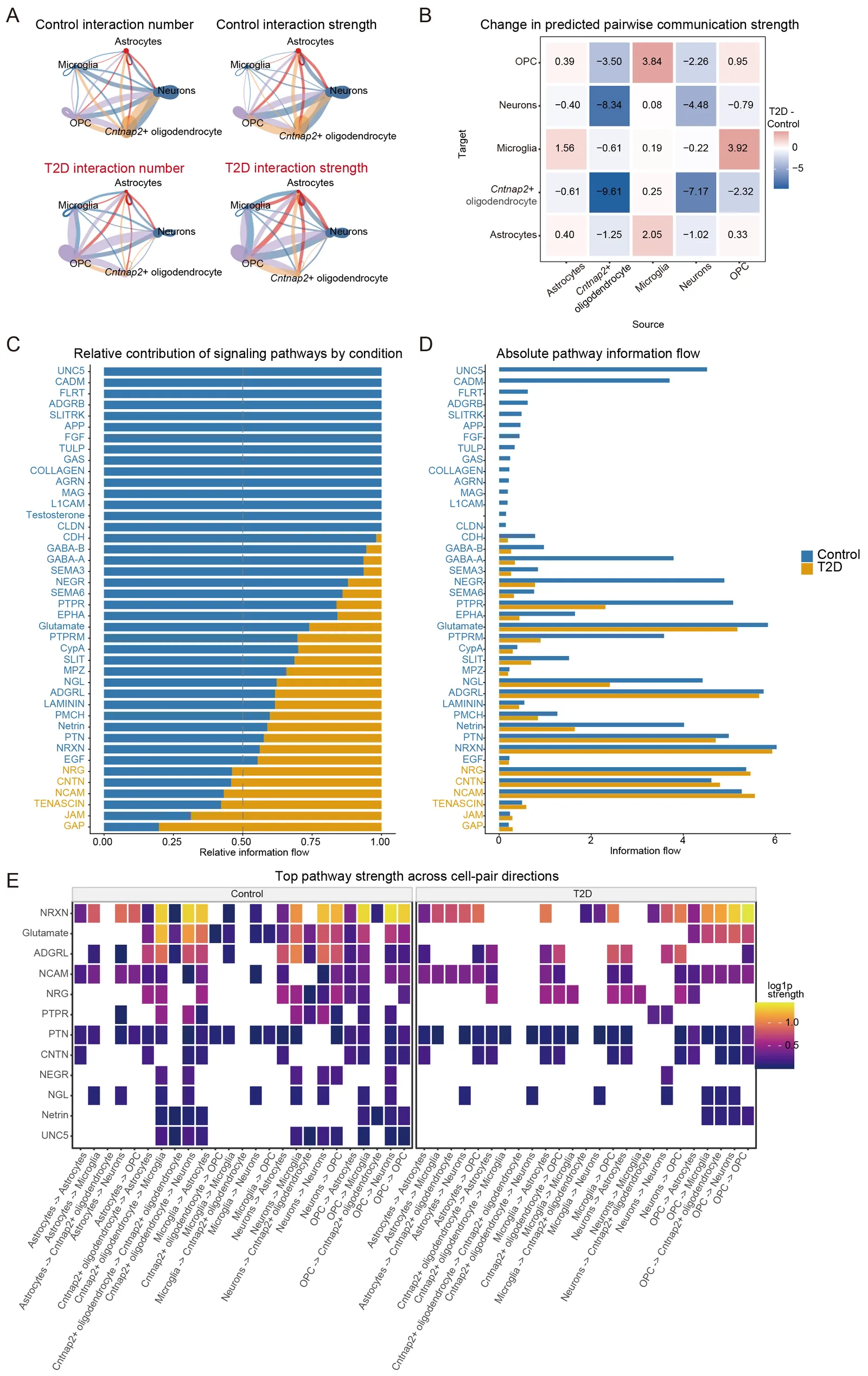
HFD/STZ-induced diabetic conditions are associated with altered predicted neuro–glial communication networks in the hypothalamus. (**A**) Circle plots showing the inferred number and strength of ligand–receptor interactions among neurons, astrocytes, microglia, OPCs, and *Cntnap2*-positive oligodendrocytes in control and T2D mice. Edge thickness represents interaction count or communication weight. (**B**) Heatmap showing differences in inferred pairwise communication strength between T2D and control groups. Values represent T2D minus control communication strength. Rows indicate target cell types, and columns indicate source cell types. Red indicates increased predicted communication under HFD/STZ-induced diabetic conditions, whereas blue indicates decreased predicted communication strength. (**C**) Relative information flow of individual signaling pathways in control and T2D mice. Pathways are ranked according to their relative contribution to the total inferred communication network. (**D**) Comparison of pathway-level information flow between control and T2D groups. Blue and orange bars indicate signaling strength in control and T2D mice, respectively. (**E**) Heatmap showing the inferred strength of selected signaling pathways across specific source–target cell-pair directions in control and HFD/STZ-treated mice. Color intensity represents log-transformed pathway communication strength.

Global analysis of the inferred communication network revealed substantial differences between control and T2D mice in both the number and overall strength of predicted cell–cell interactions (Figure 6A). Pairwise comparison further indicated that several source–target relationships were differentially represented between the two conditions (Figure 6B). In particular, predicted signaling involving neurons and *Cntnap2*-positive oligodendrocyte-lineage cells was generally reduced, including neuron-to-neuron and bidirectional neuron–*Cntnap2*-positive oligodendrocyte-lineage communication. In contrast, increased predicted communication was observed for selected glial interactions, including signaling involving microglia, OPCs, and astrocytes. These patterns suggest a redistribution of inferred intercellular signaling from selected neuronal and neuron–oligodendrocyte interactions toward specific glial communication routes under HFD/STZ-induced diabetic conditions.

Pathway-level analysis further revealed condition-associated changes in predicted signaling programs (Figure 6C,D). Several pathways involved in neuronal communication, cell adhesion, and axon-associated signaling, including UNC5, CADM, FLRT, ADGRB, SLITRK, GABA-A/GABA-B, glutamate, PTN, Netrin, and NRXN signaling, showed greater inferred information flow in control mice than in T2D mice. In contrast, several adhesion- and extracellular matrix-associated pathways, including CNTN, NCAM, TENASCIN, JAM, and GAP signaling, showed relatively greater contributions under HFD/STZ-induced diabetic conditions. Examination of selected pathways across individual source–target cell pairs further demonstrated that these pathway-level differences were distributed across specific neuronal, glial, and oligodendrocyte-lineage communication routes (Figure 6E). While computational inferences of ligand–receptor interactions reflect signaling potential rather than direct physical contact, these findings collectively demonstrate that HFD/STZ-induced metabolic disruption disrupts homeostatic neuro-glial and axon–glia communication, replacing it with an inflammatory, glial-centric network.

**Figure 7:**
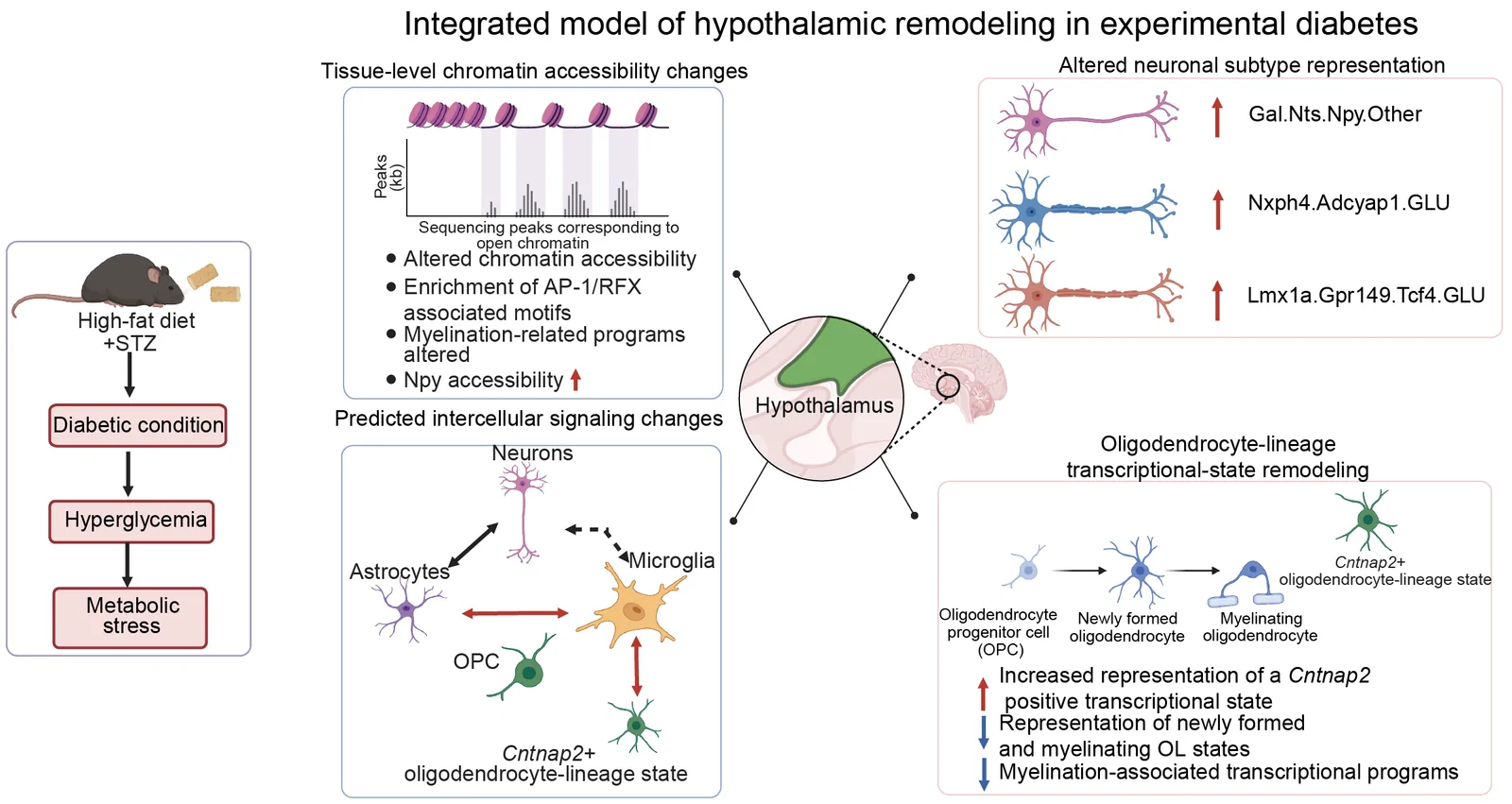
Integrated model of hypothalamic remodeling in experimental diabetes.

Together, these findings suggest that HFD/STZ-induced diabetic conditions are associated with coordinated changes in hypothalamic cellular states, transcriptional programs, and predicted intercellular signaling, involving both neuronal and oligodendrocyte-lineage populations (Figure 7).

## 4. Discussion

In this study, we integrated single-nucleus transcriptomic profiling with tissue-level chromatin accessibility profiling to uncover the multi-layered cellular and epigenomic alterations in the hypothalamus under HFD/STZ-induced diabetic conditions. Rather than being confined to individual glucose- or appetite-sensing neuronal populations, the observed changes involved multiple neuronal subtypes, oligodendrocyte-lineage differentiation states, microenvironmental neuro-glial communication, and tissue-wide chromatin accessibility [38,39,40]. These findings extend previous studies of diet-induced obesity and diabetic-associated hypothalamic neuroinflammation by providing an integrated multi-omic view of hypothalamic remodeling under diabetic metabolic stress [11,41,42].

A prominent feature of our datasets was the selective alteration of specific neuronal subclusters, particularly Gal.Nts.Npy neurons, Nxph4.Adcyap1 glutamatergic, and Lmx1a.Gpr149.Tcf4 glutamatergic neurons, rather than a uniform transcriptional response across all hypothalamic neurons. Genes expressed by these populations, including *Npy*, *Gal*, *Nts*, and *Adcyap1*, participate in the regulation of energy balance, neuroendocrine signaling, and adaptive responses to metabolic stress [43]. The preferential involvement of these neuronal populations therefore suggests that T2D conditions may selectively affect hypothalamic circuits involved in systemic metabolic regulation.

The increased expression of *Adcyap1* is of particular interest in this context. *Adcyap1* encodes pituitary adenylate cyclase-activating polypeptide (PACAP), a neuropeptide involved in autonomic, neuroendocrine, and metabolic regulation. Selective activation of PACAP-expressing neurons in the ventromedial hypothalamus has been reported to increase circulating glucose and impair glucose tolerance. Accordingly, the increased *Adcyap1* expression observed in diabetes-responsive hypothalamic neurons may reflect enhanced recruitment of autonomic or counterregulatory neuropeptide programs. Depending on the stage and severity of metabolic dysfunction, such activation could initially serve an adaptive counterregulatory role but may become maladaptive when chronically sustained [44,45,46].

Among the neuronal alterations, the *Npy* regulatory axis was supported by multiple layers of evidence. NPY is a potent hypothalamic orexigenic neuropeptide that promotes food intake and energy conservation while also influencing autonomic output, insulin sensitivity, and hepatic glucose production, thereby contributing to systemic energy and glucose homeostasis [47,48,49]. Under physiological conditions, hypothalamic *Npy* expression, particularly within the arcuate nucleus, is tightly regulated by peripheral metabolic cues including insulin and leptin [50]. In our study, increased *Npy* expression detected by single-nucleus RNA sequencing was accompanied by increased tissue-level *Npy* expression and greater chromatin accessibility at the *Npy* locus. Together, these observations suggest that enhanced *Npy* transcription under HFD/STZ-induced diabetic conditions occurs in the context of a more permissive local chromatin environment.

The increased accessibility at the *Npy* locus therefore cannot be assigned specifically to Gal.Nts.Npy neurons and may reflect both changes in cellular composition and cell-intrinsic chromatin remodeling. Nevertheless, the concordance among single-nucleus transcriptomic, tissue-level RT-qPCR, and chromatin-accessibility measurements provides convergent evidence for activation of the hypothalamic *Npy* regulatory axis. Moreover, enrichment of activity-associated transcription factor motifs, including CREB/AP-1 family motifs, within regions showing increased accessibility is consistent with the possibility that activity-dependent transcriptional regulation contributes to these changes [51]. However, direct occupancy of these transcription factors at the *Npy* locus will require experimental validation. Functionally, increased *Npy* expression may initially reflect an adaptive response to impaired insulin signaling or diabetes-associated metabolic stress [50]. Persistent activation of the NPY system, however, could potentially reinforce feeding, energy conservation, autonomic responses, and endogenous glucose production, thereby contributing to the persistence of metabolic dysregulation.

Beyond neuronal populations, our study demonstrates pronounced alterations within the oligodendrocyte lineage. T2D was associated with a reduced representation of mature, myelinating oligodendrocytes and newly formed oligodendrocytes, accompanied by the aberrant accumulation of a transcriptionally distinct, *Cntnap2*-positive oligodendrocyte-lineage population. Pseudotime analysis further showed that *Cntnap2*-positive cells preferentially occupied a distinct region of the reconstructed transcriptional trajectory, accompanied by reduced representation of canonical myelination-associated states. Because pseudotime provides a computational ordering of transcriptional similarity rather than direct evidence of developmental lineage progression, these results are most appropriately interpreted as a diabetes-associated shift in oligodendrocyte-lineage transcriptional-state distribution rather than lineage fate redirection.

The functional significance of the *Cntnap2*-positive state remains to be established. *Cntnap2* encodes contactin-associated protein-like 2, a membrane-associated cell-adhesion molecule involved in neuronal membrane organization and cell–cell interactions [52,53]. The enrichment of adhesion-related transcriptional programs within this population, together with changes in predicted signaling between neurons and *Cntnap2*-positive oligodendrocyte-lineage cells, raises the possibility that this state is associated with altered axon–glial interaction. However, neither transcriptional trajectory analysis nor ligand–receptor inference can establish impaired physical axon–glial contact or functional myelination, and these possibilities will require direct histological and physiological validation [54].

Mature oligodendrocytes provide vital metabolic coupling to underlying axons by shuttling energy substrates such as lactate via monocarboxylate transporters (MCT1) [55]. Depletion of canonical myelinating oligodendrocytes likely compromises metabolic support to long-range hypothalamic axons governing systemic glucose homeostasis and satiety, creating a localized energy crisis that exacerbates neuronal dysfunction [56,57].

Cell-to-cell communication modeling revealed a systemic wiring shift within the hypothalamic microenvironment. We observed a marked decline in neuron-centered homeostatic signaling and axon–glia interactions, parallel to elevated ligand–receptor crosstalk between microglia, OPCs, and astrocytes. This transition from physiological neuro-glial support to inflammatory glial crosstalk mirrors the localized neuroinflammatory cascades reported in non-human primate and rodent models of metabolic syndrome [58,59].

While this study offers an integrated model of HFD/STZ-induced hypothalamic remodeling, several methodological limitations point to critical avenues for future research. Firstly, because our ATAC-seq data were derived from bulk tissue, chromatin accessibility changes cannot be definitively mapped to specific cell subclusters. Future studies utilizing single-nucleus Multiome (simultaneous snRNA-seq and snATAC-seq from identical nuclei) will be essential to validate whether the increased accessibility at the *Npy* locus is exclusive to Gal.Nts.Npy neurons or reflects broader epigenomic shifts across surrounding glia [60]. Secondly, pseudotime analysis infers computational ordering rather than true developmental lineage progression. Validating the proposed transition into the *Cntnap2*^+^ state will require in vivo pulse-chase lineage tracing using inducible Cre-driver lines (e.g., Olig2-CreER or *Cntnap2*-CreER paired with reporter mice) subjected to HFD/STZ induction. Lastly, establishing the direct causality of these cell states requires functional intervention. Utilizing cell-type-specific CRISPR interference (CRISPRi) or viral-mediated knockdown of *Cntnap2* within hypothalamic oligodendrocyte lineages, combined with chemogenetic (DREADDs) manipulation of *Npy* chromatin-accessible subclusters, will clarify whether reversing this neuro-glial remodeling can restore systemic metabolic health [61]. Because HFD-only and STZ-only groups were not included, the present design does not allow the relative contributions of dietary exposure, STZ treatment, and the resulting diabetic metabolic state to be disentangled. Accordingly, the molecular alterations identified here should be interpreted as features associated with the HFD/STZ-induced diabetic condition rather than as T2D-specific effects.

In summary, our integrated analyses indicate that T2D conditions are associated with coordinated remodeling of hypothalamic neuronal states, oligodendrocyte-lineage transcriptional programs, predicted intercellular signaling, and chromatin accessibility. The convergence of increased *Npy* expression with greater accessibility at the *Npy* locus highlights a potential link between neuronal transcriptional activation and epigenomic remodeling, whereas the increased representation of a *Cntnap2*-positive oligodendrocyte-lineage state points to an additional glial component of the hypothalamic response to metabolic stress. Together, these findings support a model in which diabetes-associated hypothalamic alterations involve coordinated neuronal, glial, and chromatin-level changes rather than isolated neuronal responses, while providing specific cellular and molecular candidates for future mechanistic investigation.

## Data Availability

The original contributions presented in this study are included in the article/Supplementary Materials. Further inquiries can be directed to the corresponding author(s). The raw and processed scRNA-seq data generated in this study have been deposited in the GEO database under accession number GSE346712.

## Supplementary Materials

The following supporting information can be downloaded at: https://www.mdpi.com/article/10.3390/cells15181672/s1, Figure S1: Marker-based annotation of neuronal and oligodendrocyte-lineage subclusters in the mouse hypothalamus, Figure S2: Marker-based annotation of hypothalamic neuronal subtypes, Figure S3: Motif enrichment in T2D-gained open regions and chromatin accessibility at the *Avp* locus, Figure S4: Shared and subtype-specific transcriptional alterations in three T2D-responsive hypothalamic neuronal populations.
